# Treatment persistence and overall survival in myelofibrosis treated with ruxolitinib were not affected by the covid-19 pandemic, despite the reduced starting dose: Analysis of AIFA registries

**DOI:** 10.1007/s00277-025-06601-w

**Published:** 2025-09-20

**Authors:** Massimo Breccia, Simone Celant, Francesca Palandri, Francesco Passamonti, Pier Paolo Olimpieri, Valentina Summa, Annalisa Guarcello, Giuseppe Alberto Palumbo, Fabrizio Pane, Paola Guglielmelli, Pierluigi Zinzani, Paolo Corradini, Pierluigi Russo

**Affiliations:** 1 Department of Translational and Precision Medicine, Sapienza University, Rome, Italy; 2 AIFA Italian Medicines Agency, Rome, Italy; 3 IRCCS Azienda Ospedaliero-Universitaria di Bologna, Istituto di Ematologia “Seràgnoli”, Bologna, Italy; 4 Hematology Division, Foundation IRCCS Ca’ Granda Ospedale Maggiore Policlinico, University of Milan, Milan, Italy; 5 Hematology with BMT Unit, A.O.U., G Rodolico-San Marco University of Catania, Catania, Italy; 6 Università degli Studi di Napoli Federico II, Naples, Italy; 7 CRIMM, Center Research and Innovation of Myeloproliferative Neoplasms, DMSC, University of Florence, AOU Careggi, Florence, Italy; 8 Università degli Studi di Milano & Divisione Ematologia, Fondazione IRCCS Istituto Nazionale dei Tumori di Milano, Milano, Italy

**Keywords:** Myelofibrosis, Ruxolitinib, Starting dose, COVID-19, Overall survival

## Abstract

We analyzed the outcome of 2229 patients with myelofibrosis (MF) treated with ruxolitinib before and after the COVID-19 pandemic. Two populations of MF were defined from the AIFA web monitoring registries: the pre-COVID-19 (1703, 76.4%) and the post-COVID-19 (526, 23.6%) cohorts. The two populations were balanced using the Inversity Probability of Treatment Weighting. The median age was 69 years and 73 years in the pre- and post- COVID-19 era, respectively. There were no differences in spleen diameters at baseline prior to ruxolitinib in the two groups, but a difference in median spleen volume was noted (961 cm3 in the pre-era and 788.3 cm3 in the post-era). Overall, intermediate-2 IPSS risk were 67.2% in the pre- and 72% in the post-era, whereas the high-risk category was 32.7% and 27.9%, respectively. More patients started on a reduced dose in the post-COVID-19 era (73.5% versus 65% in the pre-era). After adjusting for the differences, an analysis of overall survival revealed no differences between the two groups (HR 0.875, *p* > 0.05). Patients who started ruxolitinib after COVID-19 had similar probability to stop treatment in the follow-up (HR 0.956, *p* > 0.05). The results indicate that COVID-19 did not affect the duration of treatment and the relative OS.

## Introduction

An outbreak of severe acute respiratory syndrome coronavirus 2 (SARS-CoV-2) started in December 2019 and then became pandemic in March 2020, with Italy being one of the first and most affected countries [1]. It has been reported that COVID-19 infection led to a particularly dismal outcome in Ph-negative myeloproliferative disorders (MPN) patients receiving immunosuppressive agents or reporting multiple comorbidities [2, 3]. The first wave of SARS-CoV-2 infection has been particularly severe in patients affected by myelofibrosis (MF) as compared to polycythemia vera (PV) or essential thrombocythemia (ET) [4]. In MF patients a high hospitalization rate and an increased rate of mortality were recorded, in particular for patients in which the treatment with *JAK2* inhibitors was discontinued at the time of hospitalization in intensive care unit [4]. A European study reported a rate of mortality in the whole MPN cohort of 28.5%, increased up to 40% for MF patients [5]. In the following waves of infection, the rate of severity and mortality decreased probably due to better critical management, vaccinations and reduced virulence of SARS-CoV-2 forms [6, 7]. Patients affected by MPN may have lower seroconversion after vaccination related to intrinsic reduced immunological competence that is related to both the hematological disease and the immunosuppressive and/or myelotoxic effects of the treatments. Different studies showed that the rate of seroconversion did not exceed 70% in MPN patients, particularly low in MF and in patients treated with ruxolitinib (RUX) [8–11]. Ruxolitinib is the first-in-class *JAK1/2* inhibitor and is the standard front-line therapy for MF-related splenomegaly and symptoms. In two phase III studies, namely the COMFORT trials that enrolled patients at intermediate-2 and high IPSS risk, it has demonstrated efficacy in reducing splenomegaly and improving constitutional symptoms. Anemia and thrombocytopenia were the most common side effects, related to myelosuppression mediated by *JAK2* inhibition [12, 13]. Indeed, *JAK1* inhibition is responsible for reduction of pro-inflammatory cytokines with improvement of disease-related symptoms but also in impaired immune function [14]. Several abnormalities both in adaptive and innate immunity were demonstrated after ruxolitinib exposure [15]. Ruxolitinib administration was not associated with reduced survival in patients affected by COVID-19 infections, except when it was discontinued, increasing the mortality compared to COVID-19 MPN patients that could continue RUX therapy during the infection [4]. To investigate the change in prognosis we analyzed a large Italian cohort of MF patients extrapolated from the AIFA web monitoring registries (wMRs).

## Materials and methods

The analysis is focused on a comparison, in terms of survival and duration of treatment, between patients who started treatment with ruxolitinib before and after the start of the COVID-19 pandemic. Two cohorts were therefore defined: (1) the pre-COVID-19 cohort, which included the patients who as of December 31 st, 2019 had at least two years of potential follow-up; (2) the post-COVID-19 cohort, which included the patients who at the data cut-off date (April 30th, 2023) had at least two years of potential follow-up, having started treatment from March 2020. The technical features of AIFA wMRs, as well as their use within the regulatory framework of drug prescriptions in Italy, have already been described in detail [16, 17].

Patients were followed for up to 24 months. Patients who had no event, were censored at day 730 since treatment start. Events included death during ruxolitinib therapy (first analysis) and ruxolitinib discontinuation (second and third analyses).

A balancing procedure was carried out between the considered subpopulations. The approach chosen for balancing is that of the Inversity Probability of Treatment Weighting (IPTW), which is based on the calculation of a system of weights, based on the probability of patients being assigned to the subpopulation to which they belong [18]. A variable is considered to be balanced if the absolute standardized mean difference (for numerical variables) or the difference between observed relative frequencies (for categorical variables) does not exceed the arbitrary but commonly used threshold of 0.1 (10%).

Survival analyzes with Kaplan-Meier curves were conducted considering the following three outcomes:

1. Overall survival, where the event is defined by the patient’s death from any cause;
2. Time to treatment (TToT) with suspensions, where the event is defined as a permanent interruption or a temporary interruption of ruxolitinib for at least 90 days.

All analyses have been conducted using the R statistical software, with special reference to the packages “survey” (for weighted KM estimators), “survminer” (for the corresponding charts) and “adjustedCurves” (for the Cox-adjusted survival analysis) [19–22].

## Results

A whole cohort of 2229 patients were analyzed: 1703 (76.4%) started ruxolitinib in the pre-COVID era and 526 (23.6%) in the post- COVID-19 era. The baseline features are shown in Table 1. Median age was 70 years (IQ range 63–76), with patients who initiated ruxolitinib therapy in the pre-pandemic era having a lower median age (69 years) compared to patients who initiated ruxolitinib therapy in the post-pandemic era (73 years). The majority of patients were aged 64–75 years (41%) with 29.5% of patients aged > 75 years. Male prevalence was observed (60%). The baseline median spleen longitudinal diameter was 20 cm (range 17–22) with a median spleen volume of 917 cm [3] (range 524.2–1560, 961 cm [3] in the pre-era and 788 cm [3] in the post-era). For the purposes of this analysis, the study population included only patients at intermediate-2 (68.3%) or high risk (31.6%) based on the IPSS model, considering the paucity of data relating to IPSS intermediate-1, in which ruxolitinib prescription has started in 2018, therefore only in post-Covid era. The IPSS risk was comparable in the pre- and post-COVID-19 era, being intermediate-2 in 67.2% of cases and 72% of cases in the two periods, respectively. The slight difference in older median age and reduced rate of IPSS high risk in the post-pandemic era it is representative of clinical reality but not attributable to bias of selection.

**Table 1:** Characteristics of patients

	Pre-CoViD-19 (*n* = 1703, 76.4%)	Post-CoViD-19 (*n* = 526, 23.6%)	Total (*n* = 2229)
Sex			
F	700 (41.1%)	191 (36.31%)	891 (39.97%)
M	1003 (58.9%)	335 (63.69%)	1338 (60.03%)
Age at treatment start			
Median (Q1-Q3)	69 (62–75)	73 (67–77)	70 (63–76)
Mean (SD)	67.3 (10.3)	71.35 (9.21)	68.25 (10.2)
Age class at treatment start			
< 50	104 (6.11%)	12 (2.28%)	116 (5.2%)
50–64	464 (27.25%)	75 (14.26%)	539 (24.18%)
64–75	679 (39.87%)	236 (44.87%)	915 (41.05%)
>=75	456 (26.78%)	203 (38.59%)	659 (29.56%)
JAK2 V617F mutation status			
Negative	362 (21.26%)	105 (19.96%)	467 (20.95%)
Positive	1341 (78.74%)	421 (80.04%)	1762 (79.05%)
Spleen’s longitudinal diameter			
Median (Q1-Q3)	20 (17–23)	18 (16–21)	20 (17–22)
Mean (SD)	19.89 (5.13)	18.83 (4.51)	19.64 (5.01)
Spleen’s transversal diameter			
Median (Q1-Q3)	10 (9–15)	10 (8–13)	10 (8-14.9)
Mean (SD)	11.75 (4.5)	10.69 (83.95)	11.5 (4.4)
Spleen’s antero-posterior diameter			
Median (Q1-Q3)	9 (6–11)	8 (6–10)	9 (6–11)
Mean (SD)	9.42 (4.02)	8.89 (3.66)	9.3 (3.95)
Spleen’s lower costal margin diameter			
Median (Q1-Q3)	10 (6–15)	7 (5–12)	9 (6–14)
Mean (SD)	10.68 (5.4)	8.93 (4.75)	10.27 (5.31)
Spleen volume			
Median (Q1-Q3)	961 (549.1-1630.4)	788.3 (434.1-1367.6)	917.3 (524.2–1560)
Mean (SD)	1304.8 (1241.7)	1074.1 (917.1)	1250.4 (1177.1)
% circulating blasts			
Median (Q1-Q3)	1 (0–2)	1 (0–2)	1 (0–2)
Mean (SD)	1.67 (1.93)	1.53 (1.99)	1.64 (1.94)
Risk category			
Intermediate − 2 (2 risk factors)	1145 (67.23%)	379 (72.05%)	1524 (68.37%)
High (> 2 risk factors)	558 (32.77%)	147 (27.95%)	705 (31.63%)
ECOG performance score			
0	620 (36.41%)	207 (39.35%)	827 (37.1%)
1	844 (49.56%)	259 (49.24%)	1103 (49.48%)
> 1	239 (14.03%)	60 (11.41%)	299 (13.41%)
Starting dose			
Full	591 (34.7%)	139 (26.43%)	730 (32.75%)
Reduced	1112 (65.3%)	387 (73.57%)	1499 (67.25%)

**Fig. 1:**
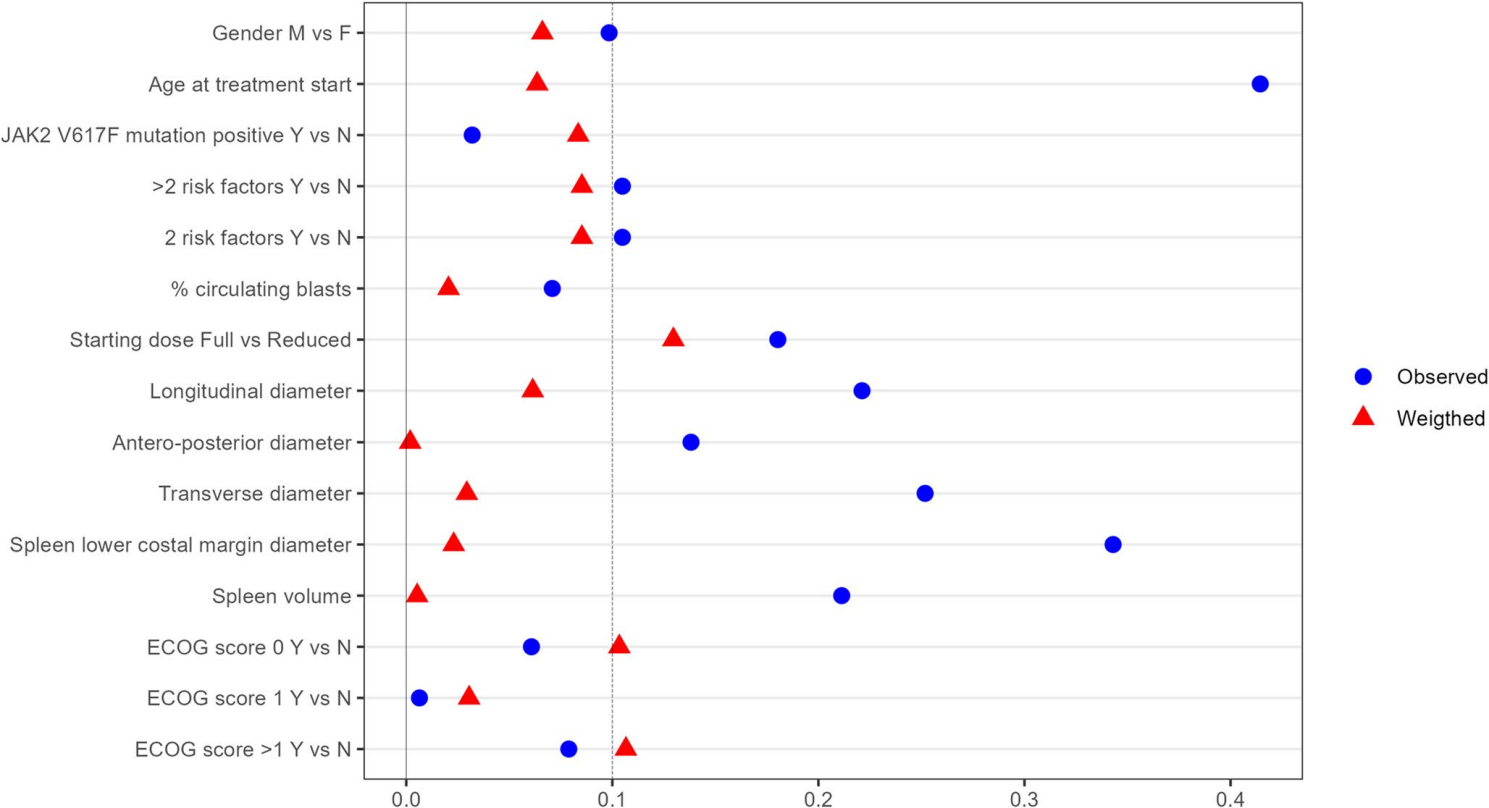
Variables balancing procedure

The results of the balancing procedures are summarized in Fig. 1: IPTW does not allow to achieve balancing in all selected covariates: prescription of a reduced starting dose, which in a previous work has been shown to be significantly associated to OS and TToT [23] is more frequent in the post-COVID − 19 era. Therefore, survival analysis has been conducted after correcting for starting dose through Cox model.

**Fig. 2:**
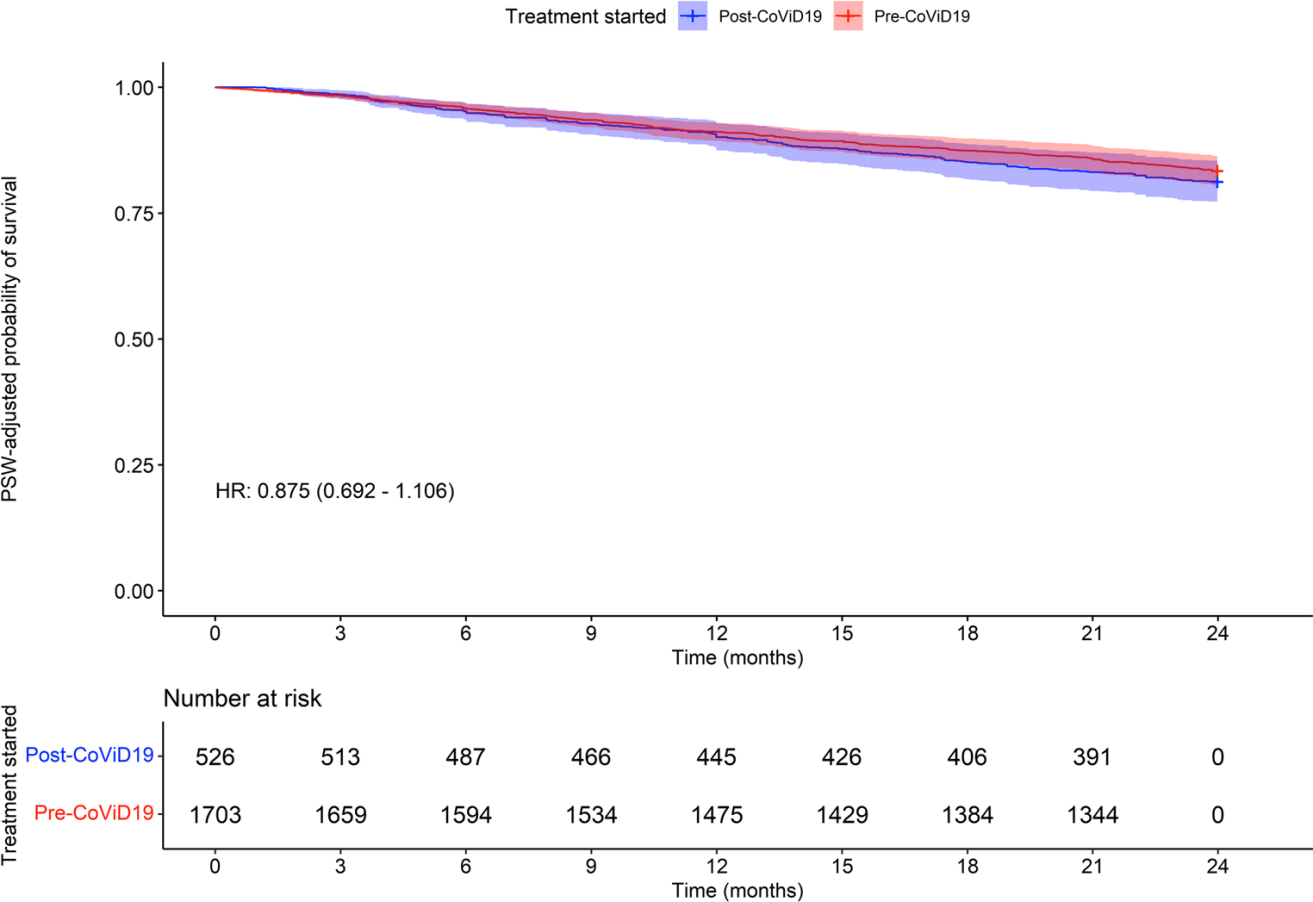
OS analysis

**Fig. 3:**
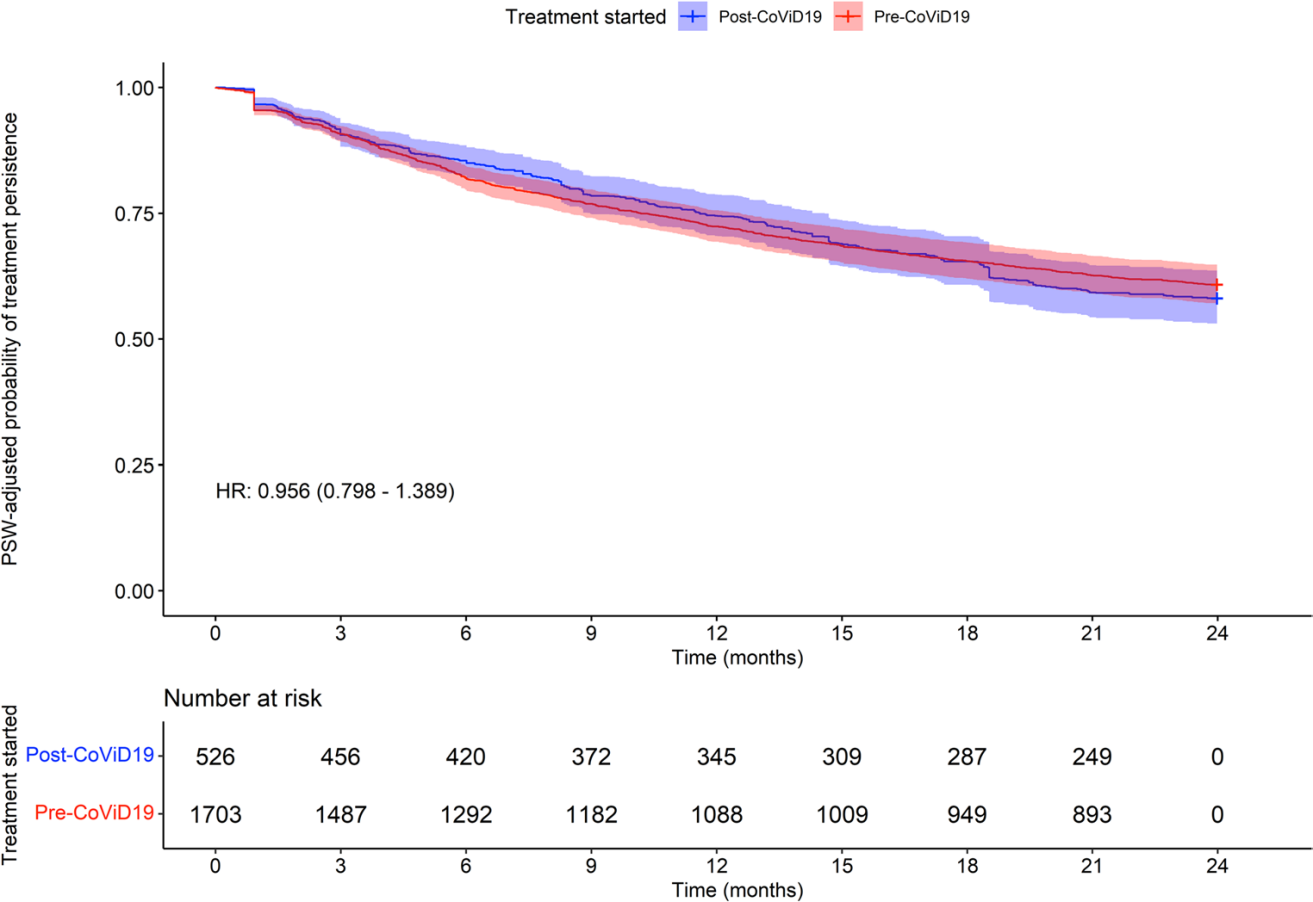
Time to treatment discontinuation analysis

Figure 2 presents a Kaplan-Meier OS analysis following feature balancing and Cox-adjustment, indicating an approximate HR of 0.875. This suggests that patients who initiated treatment in the post-COVID-19 era had comparable mortality rates during follow-up with respect to patients treated in the pre-era (*p* > 0.05). The median OS was not yet reached in either group. We also considered survival time after discontinuation: the mean number of days of survival after discontinuation was 111.4 days for pre-COVID-19 patients and 86.9 for post-COVID-19 patients. The t-test between these two means did not reject the null hypothesis of equality between the two values (*p* = 0.061), therefore the data suggest that survival after discontinuing treatment is not different between the two groups. A Cox model computed only on the data set of deceased patients, adjusted with the weights used for the balancing procedure, returned a non-significant HR (HR = 0.867, *p* = 0.234). As shown in Fig. 3, the time to treatment discontinuation was also analyzed, including, as events, all patients who discontinued ruxolitinib for a minimum period of 90 days. Patients who started treatment in the post-COVID-19 era have a similar risk of discontinuation compared to patients who started ruxolitinib earlier (HR 0.956, *p* > 0.05). In May 2022, fedratinib became available for clinical practice in Italy. Therefore, we conducted a brief analysis of patients who transitioned from ruxolitinib to fedratinib, to exclude a possible impact on outcome of this new therapeutic option. Only 9 patients started fedratinib in the post-COVID-19 pandemic (1.7%), suggesting that the potential impact on the present analyses is minimal.

## Discussion

By blocking the JAK-STAT signaling, ruxolitinib has demonstrated efficacy in reducing splenomegaly and improving constitutional symptoms in two large phase III studies (COMFORT trials) that enrolled patients at intermediate-2 and high IPSS risk [12, 13]. A recent pooled analysis has shown that early start of treatment with ruxolitinib is associated to a better outcome with increased rate of response and improved overall survival [24]. Anemia and thrombocytopenia were the most common side effects related to the myelosuppression mediated by *JAK2* inhibition. *JAK1* inhibition is associated to reduction in pro-inflammatory cytokines with consequent improvement of disease-related symptoms but also impaired immune function [14]. Moreover, several abnormalities both in adaptive and innate immunity were reported after ruxolitinib exposure [25]. For these reasons, during ruxolitinib treatment, opportunistic and atypical infections have been described together with Herpes and Zoster virus reactivations [26]. A systematic infectious screening is recommended before the start of ruxolitinib [26]. The decreased immunosurveillance associated to ruxolitinib was considered a risk factor for an increased chance of acquiring SARS-CoV-2 infection and/or developing a more severe COVID-19 syndrome in patients with MPNs [14]. Our analysis, while not reporting any drug suspensions due to possible infections, reports on large numbers that the prognosis of MF patients treated with ruxolitinib does not change compared to the pre-pandemic period, despite the effects of the pandemic and dose adjustments. Reported evidence has demonstrated that ruxolitinib treatment was not associated with reduced overall survival in patients affected by COVID-19 infections, whereas, indeed, patients who discontinued the drug had a significantly worse prognosis compared to SARS-CoV-2 positive patients that could continue ruxolitinib during the infection [4]. The ASH research collaborative registry showed that ruxolitinib discontinuation is associated with an 8.51-fold increased risk of death [27]. In a recent Italian survey, physicians declared that have started ruxolitinib in MF patients according to routine practice during the first pandemic wave. The results of the survey showed that ruxolitinib has no concomitant negative effect on COVID-19 infection and negatively influenced only 10% of MF patients with initial great disease burden [28] A recent analysis of the EPICOVIDEHA registry included 398 MPN patients: MF diagnosis, older age and baseline comorbidities, and the previous exposure to immunosuppressive agents increased the risk of death by 22%[29]. Our analysis, based on a large series of patients showed similar results in the two cohorts pre- and post-pandemic, with more older patients treated in the post-pandemic era but with less patients at high IPSS risk. After balancing the baseline features, and adjusting also for the more frequent prescription of a reduced starting dose in the post-COVID era, the results of this analysis showed that patients who started ruxolitinib, even if with an adjusted dose, during COVID-19 period, have similar risk of death in the follow-up compared to patients in pre-pandemic era. No significant difference has been observed in the risk of treatment discontinuation as well. Before starting, the seroconversion status should be obtained, but there is no indication to modify the dose in patients positive for viral infection and the complete discontinuation is associated to a worse outcome and should be discouraged. Even if ruxolitinib may impair the seroconversion after vaccination, no specific risks are reported in MF patients and all patients should receive vaccination before to start with the drug. In this analysis were considered only patients treated with ruxolitinib and only few, excluded from the study, received fedratinib. At present also momelotinib has been approved for newly diagnosed and resistant and/or intolerant patients in second line, but at the moment is really not clear the rate of discontinuation for infections and only large prospective studies will be able to clarify their role.

In conclusions, the experience gained in this pandemic year has also represented an opportunity for improvement in the treatment of MF patients. The results of this study based on a large MF population showed that SarScoV2 infection did not influenced the treatment of intermediate-2/high risk MF.

## Data Availability

No datasets were generated or analysed during the current study.

## Declarations

### Permission to reproduce material from other sources

n/a.

### Clinical trial registration

n/a.
